# Organizational stressors associated with job stress and burnout in correctional officers: a systematic review

**DOI:** 10.1186/1471-2458-13-82

**Published:** 2013-01-29

**Authors:** Caitlin Finney, Erene Stergiopoulos, Jennifer Hensel, Sarah Bonato, Carolyn S Dewa

**Affiliations:** 1 Centre for Research on Employment and Workplace Health, Centre for Addiction and Mental Health, Toronto, Canada; 2 Department of Psychiatry, University of Toronto, Toronto, Canada; 3 Library Services, Centre for Addiction and Mental Health, Toronto, Canada; 4 Head, Centre for Research on Employment and Workplace Health, Centre for Addiction and Mental Health, Full Professor, Department of Psychiatry, University of Toronto, 455 Spadina, Suite 300, Toronto, Ontario, M5S 2G8, Canada

## Abstract

**Background:**

In adult correctional facilities, correctional officers (COs) are responsible for the safety and security of the facility in addition to aiding in offender rehabilitation and preventing recidivism. COs experience higher rates of job stress and burnout that stem from organizational stressors, leading to negative outcomes for not only the CO but the organization as well. Effective interventions could aim at targeting organizational stressors in order to reduce these negative outcomes as well as COs’ job stress and burnout. This paper fills a gap in the organizational stress literature among COs by systematically reviewing the relationship between organizational stressors and CO stress and burnout in adult correctional facilities. In doing so, the present review identifies areas that organizational interventions can target in order to reduce CO job stress and burnout.

**Methods:**

A systematic search of the literature was conducted using Medline, PsycINFO, Criminal Justice Abstracts, and Sociological Abstracts. All retrieved articles were independently screened based on criteria developed a priori. All included articles underwent quality assessment. Organizational stressors were categorized according to Cooper and Marshall’s (1976) model of job stress.

**Results:**

The systematic review yielded 8 studies that met all inclusion and quality assessment criteria. The five categories of organizational stressors among correctional officers are: stressors intrinsic to the job, role in the organization, rewards at work, supervisory relationships at work and the organizational structure and climate. The organizational structure and climate was demonstrated to have the most consistent relationship with CO job stress and burnout.

**Conclusions:**

The results of this review indicate that the organizational structure and climate of correctional institutions has the most consistent relationship with COs’ job stress and burnout. Limitations of the studies reviewed include the cross-sectional design and the use of varying measures for organizational stressors. The results of this review indicate that interventions should aim to improve the organizational structure and climate of the correctional facility by improving communication between management and COs.

## Introduction

Workplace stress and burnout affects between 19% and 30% of employees in the general working population [1-3]. Job stress is the psychological distress or strain that arises from both individual and organizational stressors in the workplace [1,4]. Long term job stress can lead to burnout in the workplace and is characterized by feelings of exhaustion, cynicism, detachment, ineffectiveness and lack of personal accomplishment [5]. Both job stress and burnout can result in employees with decreased organizational commitment and associated lower productivity [5]. Over the past three decades, a large body of research has examined the factors contributing to job stress and burnout and there is a growing need to critically examine the organizational stressors specifically, in order to create healthy employees and work environments [1,6-8].

Correctional facilities employees are potentially exposed to a greater number of on the job risk factors because they house a population against their will with the mission of “contribut[ing] to public safety by actively encouraging and assisting offenders to become law-abiding citizens, while exercising reasonable, safe, secure and human control” [9]. Front-line correctional officers (COs) are the employees who are responsible for keeping the facility safe and secure, maintaining the population of inmates and helping to facilitate their rehabilitation [10-12]. Given the nature of correctional facilities and the service that is provided, the organizations that operate the facilities are characterized by “strict hierarchies…and pervasive bureaucrac[ies]” [13]. The organizational structure of corrections and, consequently, the hierarchical relationship between management and staff can cause stress and job dissatisfaction [13]. Within correctional facilities (e.g. prisons, jails), it is estimated that 37% of COs experience job stress and burnout [2]. This is higher than the estimated 19-30% in the general working population. COs who experience symptoms of stress and burnout have the potential to show a lack of motivation and a lack of commitment, resulting not only in decreased organizational commitment [14], but also in an increase in counter-productive attitudes and behaviors. Counter-productive attitudes and behaviors compromise the safety and security of the correctional facility as well as inmate rehabilitation. An example of a counter-productive behavior is aiding and abetting inmates in carrying out criminal behavior from within the prison [15].

In recent years, COs have been facing increasingly high rates of workplace stress [16] which can produce a detrimental impact on the safety and security within correctional facilities. It is therefore important to examine the organizational stressors that are associated with CO stress and burnout. In their study of police officers, Crank et al. (1995) reported that examining organizational level stressors was of importance due to “their ability to overwhelm otherwise beneficial individual-level characteristics” [17]. That is to say that individual-level characteristics can moderate the effects of job stress, however, even those beneficial characteristics become less helpful under conditions of enduring or overwhelming organizational stressors [17]. An awareness of the organizational stressors impacting employee workplace stress and burnout would enable the identification of organizational interventions that can more accurately target these areas, thereby reducing stress and burnout [7].

A previous literature review by Shaufeli and Peeters (2000) has detailed some of the organizational stressors that COs face [18]. This review, however, organized their results by type of facility rather than type of staff. In addition, more recent research has highlighted several new organizational stressors that were not mentioned in this review.

Cullen et al. (1985) have noted that COs need to be conceptualized as unique from other employees within correctional facilities since COs “work in an unusual social setting and have an unusual technical task” [4]. In addition, a recent meta-analysis of work-related stress in COs, by Dowden and Tellier (2004), called for a more detailed analysis of job position as it may play a significant role in moderating the effects of job stress [19]. In support of this hypothesis, studies have demonstrated that different types of correctional employees have varying levels of job stress and burnout [19-22]. For example, COs have higher levels of job stress than both supervisory COs [22] and employees who work in non-custody positions within the correctional facility [20]. Correctional employees are also exposed to different organizational stressors within the correctional facility. Studies have found differences between COs and other correctional employees on measures of role strain, perception of intrinsic and extrinsic rewards, job satisfaction [23] and organizational commitment [24].

More recent research on CO stress has indicated several organizational stressors that were not previously examined by Shaufeli and Peeters (2000) including, but not limited to, organizational climate, resources, rewards and quality of supervision [18]. Given the differences in experienced job stress and burnout, interventions may need to be employee group specific and address different areas of the organization depending on the targeted employee group. Since they presented an aggregate review, the results presented by Schaufeli and Peeters (2000) may have been confounded by the use of studies that combined various correctional employees in their samples [18].

The purpose of this paper is to review the scientific literature on job stress and burnout in COs employed in adult correctional facilities in order to examine organizational stressors that are related to CO job stress and burnout. This paper fills a gap in the organizational stress literature among COs by focusing on a specific group within the facility and examining organizational stressors that were not previously identified. This review marks the first step to identifying the areas within correctional organizations that can be targeted by interventions in order to promote a healthy and productive workplace for COs.

## Background

### COs and the facilities they work in

Within correctional facilities, COs have the primary responsibility of maintaining safety and security within the walls of the institution by closely monitoring, supervising and managing the inmates [10-12]. COs also have the task of aiding offender rehabilitation, preparing them for re-entry into society and ultimately contributing to the prevention of recidivism [10,11]. Despite consensus in the literature on the role of COs, numerous terms are used to describe this position. They include: corrections officers (North America and New Zealand) [10-12], agentes penetenciarios (Brazil) [25], prison officer (Britain, Australia (22), Finland) [26,27], surveillants (France) [28], personnel de interior y vigilancia (Spain) [29]. For the purposes of this paper, CO will encompass all of these terms.

Types of correctional facilities include prisons and jails, all of which have varying levels of security ranging from minimum to medium to maximum [30]. In countries like the United States, prisons are operated by either the state or federal government and are used to house offenders who have received a sentence of over one year due to their commission of a more serious crime [31]. Jails in the United States, on the other hand, are operated by municipal governments and are used to house offenders who are awaiting trial or those who have committed less serious offences and have a sentence of less than one year [31]. The United States also has a two-tiered correction system where facilities are operated by the government, mentioned above, as well as by for-profit private companies.

In countries like France and South Korea all prisons and jails are operated by the federal government [32,33]. Prisons and jails, similar to the United States, are used to house offenders who have received longer sentences due to the more serious nature of their crimes and short stay offenders who have committed lesser crimes respectively [32,33].

### Stress and burnout

Stress is the psychological strain or distress resulting from exposure to unusual or demanding situations, known as stressors [4]. Occupational stress, specifically, is the response to organizational stressors in the workplace environment that pose “a perceived threat to an individual’s well-being or safety” [1,4]. In addition to organizational factors, individual level factors have also been implicated in stress outcomes, both as contributing factors as well as moderators of stress [17].

Long term stress can lead to burnout which is conceptualized as a “psychological syndrome in response to chronic interpersonal stressors on the job” [5] which arises due to an imbalance between the demands placed on individuals and their ability to cope [34]. This syndrome is characterized by feelings of exhaustion, cynicism, detachment, ineffectiveness and a personal lack of accomplishment [5].

### Stressors associated with stress and burnout

A large body of literature has pointed to the multiple factors that have been implicated in stress and burnout among the general working population. Specifically, stress and burnout stem from a combination of individual risk factors and organizational stressors. Organizational stressors such as work overload, role conflict, under-promotion and level of participation interact with individual factors such as personality and family problems to create mental and physical ill health in employees [1]. Job stress can also result from an imbalance between the demands placed on individuals and their ability to cope [35] or an imbalance between employees’ efforts on the job and the subsequent rewards they receive [36].

Cooper and Marshall’s (1976) model of job stress conceptualizes five categories of workplace-specific sources of stress within an organization [1]. This model has been applied to a wide variety of employees including: social workers [37], police, nurses and firefighters [38]. The five categories of job stress as specified in this model are used to conceptualize organizational stressors in the current literature review.

The first category, *stressors that are intrinsic to the job,* describes factors that increase the difficulty and complexity of the duties that workers, in this case COs, must perform. In addition, this category also describes the factors that make a workload too heavy for the employee to handle [1,39,40].

The second category is *role within the organization* and is used to reflect role ambiguity and role conflict [1]. Role ambiguity arises when the duties and expectations placed on the employee are unclear [4,41,42]. Role conflict occurs when there are conflicting demands placed on the employee [1]. Among COs, this can be seen through the expectation to exercise professionalism within a bureaucratic correctional system where COs do not have the authority to do so [4]. For example, COs must often maintain security through informal interactions with inmates that may not comply with the written rules of the correctional facility [4].

The third category of work-specific stressors is *career development* which is used to encompass the factors affecting the future of an employee within an organization like promotion, job security and ambition [1]. The fourth category, *relationships at work*, describes the interactions that occur between the employee and their subordinates, co-workers and supervisors [1].

The *organization’s structure and climate*, the final category, is used to describe how the structure of the organization affects the employee. Examples of organizational structure include employees’ degree of decision latitude, organizational politics and communication between the organization and staff [1].

### Outcomes of stress and burnout

Within the general working population, long term stress and burnout in the workforce can result in a negative overall mood [6], physical ill health, job dissatisfaction and increased substance abuse [1]. Occupational stress can also result in a decrease in organizational commitment and avoidance behaviors at work, such as absenteeism and sick day use [6]. In addition, burnout can cause lower productivity and ineffectiveness at work [5].

Among correctional employees, stress and burnout can also lead to negative personal, social and work outcomes. However, these effects are more pronounced in this population when compared with the general working population, in part attributable to higher rates of stress and burnout [16]. In correctional staff, work stress and burnout have been shown to be associated with decreased life satisfaction [14], internal withdrawal [13], inability to cope with traumatic experiences [13], decreased physical health [43] and increased substance use [13]. A decrease in positive social interaction and relationships [14] and an increase in work-family conflict [43] may also be related to correctional employee stress and burnout. From an organizational standpoint, correctional employee stress and burnout manifests itself in decreased job involvement [44], lower job satisfaction [44], reduced organizational commitment [19], negative safety outcomes [43], an increase in turnover [14], increased absenteeism [19] and higher use of sick days [43].

This paper fills a gap in the organizational stress literature among correctional officers by systematically reviewing the way in which organizational stressors are related to CO job stress and burnout in adult correctional facilities. In doing so, the present review identifies the areas that organizational interventions can target in order to reduce CO stress and burnout.

## Methods

### Literature search

Four electronic databases were used in this systematic review: Medline, PsycINFO, Criminal Justice Abstracts and Sociological Abstracts. All searches were conducted on May 31, 2012. This study used existing literature and therefore, did not involve living subjects. Consequently, it did not require Research Ethics Board review. The search strategies, as outlined in the Appendix, included key terms such as correction officer, prison guard, job stress and burnout. All searches were limited to results from 1999 to 2012, in order to yield the most current studies that have not appeared in previous reviews [18]. Using selection criteria developed *a priori*, presented in Table 1, raters CF and ES independently screened the resulting titles, abstracts and full text articles.

**Table 1:** Selection criteria

	
**Inclusion Criteria**	
Yes	**Diagnosis** (one of the following must be checked off as a ‘yes’)
□	□ Burnout (shows symptoms as determined by a valid psychometric measurement and/or biomedical measure)
	□ Stress (shows symptoms as determined by a valid psychometric measurement and/or biomedical measure)
	**Correlation** (both must be checked off as a ‘yes’)
□	□ Must measure correlates of stress and/or burnout
	□ Correlates must be organizationally-based
	**Outcome** (must be checked off as ‘yes’)
□	□ Description of the how the stressor is correlated to job stress or burnout
**Exclusion Criteria**	
Yes	**Sample Population** (any of the following are grounds for exclusion)
□	□ A group that does not consist of front line correctional officers
	□ A group not employed in an adult correctional facility (ie. juvenile detention center, juvenile correctional facility, treatment facility, community corrections, probation office, parole office)
	**No Outcomes** (the following is grounds for exclusion)
□	□ Describes offender outcomes, prisoner mental health, prisoner stress
	□ No outcomes about the sample population
	**Type of article** (any of the following are grounds for exclusion)
□	□ Non peer-reviewed article
	□ Book review
	□ Editorial
	□ Dissertation

### Eligibility criteria

In this review, job stress was defined as the “psychological discomfort or tension” [4] that results from exposure to organizational stressors in the workplace environment [1]. Burnout was defined as the subjective experience that results from chronic stressors from the workplace and is characterized by feelings of exhaustion, cynicism, detachment, ineffectiveness and a personal lack of accomplishment [5]. The studies resulting from the database searches were screened on the basis of (a) diagnosis of stress or burnout using a validated measure, (b) description of the organizational correlates of job stress or burnout (with or without a validated measure), (c) study sample of COs and (d) employment within an adult correctional facility. Studies in English, French, Spanish, Portuguese, Italian, Greek, Polish and Croatian were eligible for inclusion. This review does not break down findings based on facility type due to the small number of included studies and some evidence that the environmental differences in correctional facilities do not have a significant impact on CO job stress [19].

Studies were excluded on the basis of (a) lack of empirical data, (b) solely a description of offender outcomes, for example organizational stressors that impact inmate stress, and (c) solely a description of non-organizational correlates of stress, for example studies examining the relationship between age and stress. Studies were also excluded if they used a sample of correctional employees, in addition to front-line COs, and did not separate them out in the analysis of their results. Examples of correctional employees who are not front-line officers include, but are not limited to: supervisory correctional officers, case managers, medical staff, industry staff, food service workers, supervisory staff, wardens and treatment officers.

After titles were screened according to the eligibility criteria, relevant abstracts were retrieved and screened using the same criteria. Full-text articles were then retrieved and evaluated based on the inclusion and exclusion criteria outlined in Table 1. The four electronic database searches revealed a total number of 313 articles for examination, excluding duplicates.

### Assessment of methodological quality

An 8-item quality assessment checklist was developed *a priori* to assess the methodological quality of the included studies adapted from a previous checklist used by Stergiopoulos et al. (2011) [45]. Assessment questions examined study design, methods, measurements, analysis and data presentation. Methodological quality was independently assessed by raters CF and ES. Any discrepancies with regards to the ratings were discussed and agreement was reached between both raters. Studies meeting all assessment criteria were rated as “excellent” while studies that met at least 4 out of the 8 criteria were rated as “good” as outlined in Additional file 1.

### Stressor classification system

Five main categories of work stressors were outlined in Cooper and Marshall’s (1976) model of work related stress [1]. These broad categories have been used to conceptualize organizational stressors in a broad range of literature on occupational stress [38,46,47] and were adopted as the stressor classification system for this review. The five categories of work stressors in Cooper and Marshall’s model are stressors intrinsic to the job, role in the organization, career development, relationship at work and organizational structure and climate [1]. For the purposes of this paper, the classification *rewards at work* will simultaneously replace and encompass the original category *career development*. It has been shown that the term “organizational rewards” is more able to accommodate for not only career developments, like salary, but also other rewards specific to the job, including intrinsic rewards like pride [8]. In addition, *relationships at work* has been narrowed in this paper to *supervisory relationships at work* in order to reflect the importance, as evidenced by the literature included in this review, of COs' relationships specifically with supervisors. Table 2 lists the five categories used in this paper and the resulting classification of the various stressors identified by the included studies.

**Table 2:** Organizational classification of stressors

**Organizational classification**	**Stressors**
Intrinsic to Job	Work overload
	Overtime
	Training
	Overcrowding
	Participation
	Skill Utilization
	Professional worth
	Work-related tasks outside of the facility
	Limited resources
	Understaffing
	Daily tasks
Role in Organization	Role problems
Rewards	Perceived intrinsic rewards
	Salary
	Opportunity
Supervisory Relationships at Work	Negative interaction with supervisors
	Perceived supervisory influence
	Leadership issues
	Quality of supervision
	Supervisory support
Organizational Structure and Climate	Organizational support
	Organizational justice
	Organizational climate
	Administrative strengths

## Results

**Figure 1:**
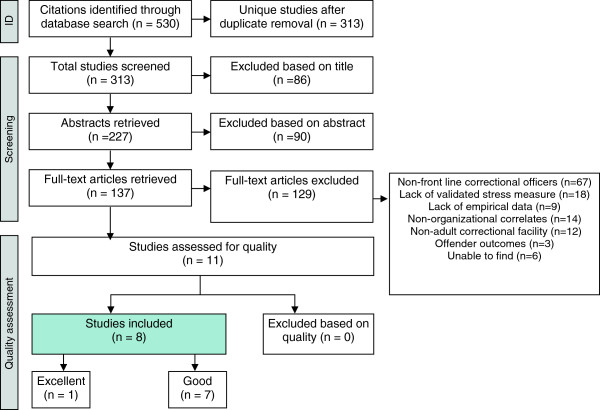
Literature search results and inclusion–exclusion process.

The systematic search of four electronic databases, Medline, PsycINFO, Criminal Justice Abstracts and Sociological Abstracts generated a total result of 313 articles, excluding duplicates. The two raters independently screened all titles and abstracts and reviewed 137 full-text articles. The inter-rater reliability between CF and ES was 0.67. Of the 137 full-text articles reviewed, 129 articles were excluded mainly due to (a) COs from adult correctional facilities were not a uniquely identified group (e.g. case workers or juvenile facility COs); (b) lack of a validated stress or burnout measure, (c) lack of any empirical data, (d) non-organizational correlates of job stress; (e) outcomes that were not for COs. In addition, there were six studies that the authors were unable to locate, despite their efforts to contact the corresponding authors of the papers and the publishing journal. Figure 1 outlines the process of inclusion and exclusion.

### Methodological quality

Eight studies met all the inclusion criteria and were subsequently assessed for methodological quality. One study that met all the eligibility criteria received a rating of “excellent”. The remaining seven studies met at least 50% of the assessment criteria and received ratings of “good”. There were no studies that received a rating of “fair” due to meeting less than 50% of the assessment criteria. As such, based on the methodological quality, all 8 of the studies were included.

### Characteristics of included studies

This systematic review included studies from 1999 to 2012, as a previous literature review examined the literature on CO stress and burnout prior to 1999 [18]. Six of the included studies originated from the United States and the remaining two from South Korea and France. The eight included studies were all cross-sectional with samples mainly consisting of male Caucasians with high-school level education employed in adult correctional facilities; both prisons and jails. All correctional facilities were public rather than private (Table 3). Table 4 outlines the organizational stressors examined by each of the eight included studies.

**Table 3:** Demographic variables

**Reference**	**Country**	**Facility type**	**Average age**	**Percentage male**	**Racial majority**	**Average tenure**	**Average education level**
Armstrong & Griffin (2004)	United States of America	Maximum security facility	35.6	76.7%	Caucasian	4 years or less	N/A
Moon & Maxwell (2004)	South Korea	Prison or jail	N/A	77.3%	N/A	N/A	Post-secondary
Castle & Martin (2006)	United States of America	County jails	38	72.4%	Caucasian	7 years	High school or equivalent
Griffin (2006)	United States of America	State prisons	34.5	75.3%	Caucasian	4 years or less	High school or equivalent
Neveu (2007)	France	Level 1	33.5	87%	N/A	8 years	High school or equivalent
Castle (2008)	United States of America	County jails	38	72.4%	Caucasian	7 years	High school or equivalent
Taxman & Gordon (2009)	United States of America	Level 2: low-medium security	43.44	68%	Caucasian	6.9 years	High school or equivalent
Summerlin, Oehme, Stern & Valentine (2010)	United States of America	N/A	N/A	N/A	N/A	N/A	N/A

**Table 4:** Organizational stressors and outcomes

**Reference**	**Sample**	**Stress/Burnout instrument**	**Stressors**	**Outcomes**
Armstrong & Griffin (2004)	3,794 COs	Five items (Crank, Regoli, Hewitt & Culbertson, 1995)	Role problems (6 items, Hepburn & Knepper, 1993)	Job stress
			Perceived intrinsic rewards (6 items, Mottaz, 1981)	
			Quality of supervision (7 items, Saylor 1981)	
			Organizational support (3 items, Eisenberger et al., 1986)	
Moon & Maxwell (2004)	318 COs	Work stress (5 items) (Cullen, Link, Wolfe & Frank, 1985)	Work overload (5 items, validated)	Job stress
			Supervisory support (5 items, validated)	
Castle & Martin (2006)	373 COs	Occupational stress (6 items) (Cullen, Link, Wolfe & Frank, 1985)	Working overtime (1 item)	Job stress
		Prison Social Climate Survey Adaptation (5 items) (Saylor, 1983)	Inmate overcrowding (1 item)	
			Levels of staffing (1 item)	
			Training prior to employment (1 item)	
			Role problems (5 items, not validated)	
			Opportunity for promotion (not reported)	
			Salary (1 item, annual salary)	
			Supervisory support (6 items, Cullen et al. 1985)	
			Administrative strengths (10 items, Saylor, 1984)	
Griffin (2006)	2,576 COs	Five items (Crank, Regoli, Hewitt & Culbertson, 1995)	Quality of supervision (7 items, Saylor 1981)	Job stress
			Organizational support (3 items, Eisenberger et al., 1986)	
Neveu (2007)	707 COs	Maslach Burnout Inventory – Human Service Survey (MBI-HSS: 22 items) (Dion & Tessier, 1994, Maslach, Jackson & Leiter, 1996)	Participation (3 items, validated)	Burnout
			Skill utilization (4 items, validated)	
			Professional worth (4 items, validated)	
Castle (2008)	373 COs	Job stress scale and general stress scale (Dowden & Tellier, 2004)	Role problems (5 items, not validated)	Job stress
			Opportunity for promotion (not reported)	
			Supervisory support (not reported)	
			Administrative strengths (not reported)	
Taxman & Gordon (2009)	1,231 COs	Three items (Peters, O’Connor & Rudolf, 1980)	Organizational justice (13 items, Sweeny & McFarlin, 1997)	Job stress
Summerlin et al. (2010)	133 COs	Operational Police Stress Questionnaire (PSQ-Op: 20 items) and Organizational Police Stress Questionnaire (PSQ-Org: 20 items) (McCreary & Thompson, 2006)	Levels of staffing (1 item, PSQ-Org, McCreary & Thompson, 2006)	Job stress
			Daily operational tasks (PSQ-Org, McCreary & Thompson, 2006)	
			Work-related activities outside of correctional facility (PSQ-Org, McCreary & Thompson, 2006)	
			Style of leadership (PSQ-Org, McCreary & Thompson, 2006)	

### Stressors intrinsic to the job

Three of the included studies demonstrated that stressors intrinsic to the job were associated with job-related stress. The stressors identified in this category were: working overtime, inmate overcrowding, levels of staffing, training, tasks within and outside of the correctional facility and resources available.

Moon and Maxwell (2004) studied work overload, using a validated measure that examined COs' perceptions about the difficulty and complexity of demands at work [40]. Examples of these items include number of assignments, types of tasks and the expectations of the quality of work. The authors found that workload was significantly associated with stress. Working overtime, measured with an unreported measure, on the other hand, was not significantly associated with any kind of stress, neither general nor occupational [42]. Similarly, inmate overcrowding, measured by examining the daily number of the inmate population, within a facility was also not a significant predictor of either occupational or general stress [42]. It is worthy to note, however, that the study acknowledged that this result was surprising and may not be typical due to the fact that 60% of the COs used in the sample stated that the facility was operating at or under capacity [42].

Levels of staffing and their effect on job stress resulted in inconsistent findings. One study, using a validated measure, indicated that levels of staffing were significantly negatively associated with levels of COs' stress [48]. Another study, that examined levels of staffing by looking at the average daily population of inmates, however, showed that understaffed facilities did not have a significant impact on CO's stress [42]. Again, it is worthy to note that over half of the COs sampled in this study reported that the facility was not operating over capacity and therefore may have impacted this result.

Among COs, training prior to employment is seen as a way to prepare them for the situations that they might face within the correctional facility [42]. Pre-job training, using a measure that was not reported, was also shown to be significantly positively associated with general stress but not occupational stress [42]. However, the authors note that this was an unexpected finding that may have been confounded by the fact that there was no indication as to the amount of training that their sample of COs received before employment [42].

COs have responsibilities both inside and outside of the correctional facility. Inside a correctional facility, their daily operational tasks, characterized as professional responsibilities outside of inmate supervision, include things like completing paperwork and addressing negative comments from the public. CO professional duties outside of the correctional facility include activities like escorting inmates to court hearings. One study, using a validated measure with items such as “Too much computer work”, indicated that daily operational tasks are not significantly associated with job stress. On the other hand, duties occurring outside, measured by validated items such as “Dealing with the court system”, of the correctional facility were a significant predictor of job stress [48].

The study by Summerlin et al. (2010) revealed that 42.1% of COs experienced high levels of stress when they did not have the resources necessary to perform their job optimally [48]. The resources outlined in this study were also measured using a validated measure and included items like “Lack of resources” and “Inadequate equipment”, and were demonstrated to have a significant impact on COs’ job stress [48].

### Role in organization

Role problems are characterized by role ambiguity and role conflict. Among COs, role ambiguity arises when the expectations placed on a CO are clear however the means by which they are to achieve these expectations remains unclear [41,42]. Role ambiguity can also be seen through an increase in misinterpretations of colleagues’ actions due to the fact that COs are often forced to work alone rather than collaboratively [4]. Role conflict occurs when COs are expected to act professionally within certain strict regulations and hierarchies of the correctional facility however do not have the formal authority to do so. Often, the management of the inmate population requires flexibility on the part of the COs as well as the use of informal social interactions that may not comply with the written rules and regulations, in order to carry out their central task of maintaining safety and security [4,42]. Studies looking at the association between role problems and CO stress had inconsistent findings. Castle (2008) measured role problems using a measure that was not validated that included statements such as “What I actually do often conflicts with what policy dictates what I do at work” and “My job duties and work objectives are unclear”. The author showed that role problems were not significantly associated with job stress [41]. On the other hand, another study, using the same measure, indicated that role problems were a significant indicator of stress [42]. A third study, using a validated measure with items such as “One of the problems here is that it’s never very clear as to who is responsible for doing different jobs”, also found that role problems were significantly associated with job stress [49]. Castle, who did not find a relationship between role problems and stress, did note some possibly contributing limitations of their study [41].

In addition to role problems, personal resources were measured by one of the studies. There are three characteristics of personal resources: participation, skill utilization and professional worth. Participation is the COs’ ability to influence organizational decisions and is measured using a validated scale with statements such as “A prison officer is here to keep his mouth shut, period” [50]. Skill utilization, a CO’s belief that they are able to use more than a basic skill set to perform their duties, for example having to creatively problem solve, is measured using a validated measure and includes items such as “My skills are recognized and utilized” [50]. Professional worth, as measured using validated items such as “To be a prison guard is a real job”, is the self-esteem that results from a CO’s professional achievements within the facility [50]. The study showed that a reduction in these three personal resources was significantly related to high levels of burnout, including the symptoms of emotional exhaustion, depersonalization and reduced personal accomplishment [50].

### Rewards at work

Rewards at work can be both intrinsic and extrinsic. Intrinsic rewards refer to the personal fulfillment that COs experience as a result of their efforts. An extrinsic reward is the organizational recognition that COs receive for their efforts, such as an increase in salary or the opportunity for promotion [41,42,49]. A lack of intrinsic rewards, as demonstrated by one study using validated items such as “I have the chance to do things that make use of my abilities”, had a significant association with CO stress and health problems [49]. On the other hand, the relationship between the type of extrinsic reward and its relationship with job stress had differing results. Two studies indicated that opportunity for promotion, using a measure that was not reported by the authors, within an organization was not a significant predictor of job stress [41,42]. On the other hand, one study determined that salary, measured using the CO’s annual salary, was significantly negatively associated with occupational stress [42].

### Supervisory relationships at work

Quality of supervision and perceived supervisory support was measured by four of the included studies. Quality of supervision is seen through the communication between supervisors and COs. Communication includes ongoing feedback on job performance and the ability of COs to express their opinions, questions and concerns of job-related matters to supervisors [49,51]. Perceived supervisory support is seen through a supervisor’s encouragement of COs to work effectively, to be proud of their accomplishments in the workplace and to have self-esteem [40-42]. In addition, supervisory support can also be seen in COs' perceptions of a supervisor’s emotional support of the CO, their instrumental support (i.e. extra resources and assistance to complete tasks) and in low levels of conflict and hostility between COs and their supervisors [16].

Quality of supervision, specifically, was examined by two of the studies. Both studies yielded disparate findings. One study measured this factor using a validated measure with items such as “I am free to disagree with my supervisor”. This study showed that a lower quality of supervision was significantly related to job stress [51]. The second study, using the same scale, however, revealed that the quality of supervision was not significantly associated with job stress [49]. The authors of the second study state that this result may be due to the impact of increased social support from co-workers and the subsequent decline in the need for supervisory feedback and communication [49].

Supervisory support was measured by four of the included studies. One study, using validated items that included statements about negative interactions with supervisors, indicated that supervisory support was significantly related to job stress [40]. A second study, using an unreported measure, also found that supervisory support was significantly related to job stress [41]. In a different finding, one study, using validated items such as “My supervisors often encourage the people I work with if they do their job well”, found that supervisory support was negatively associated with job stress, in other words, as supervisory support increased, job stress decreased. The association between these two variables, however, was not statistically significant [42]. Despite the fact that the author did not provide an explanation for these findings, it is worth noting that this study did mention some limitations including that 60% of their sample reported that the facility was operating at or below capacity, and that their response rate was only 18% [42].

Another aspect of supervisory relationships in the workplace is style of leadership displayed by the supervisor which was examined in one study. Leadership styles were examined using a validated measure that included items such as “Inconsistent leadership style” and “Unequal sharing of work responsibilities”. The study concluded that these aspects of a leader’s style were significantly negatively associated with job stress [48].

### Organizational structure and climate

The structure and climate of an organization are determined by its administrative strengths: organi-zational support and organizational justice. Administrative strengths within the organization of corrections include factors like a clear outline of policies and authorities within the institution and the ability to participate in decision-making. Four of the included studies measured administrative strengths. One study, using a validated measure with statements such as “The information I get through policies and the administration helps me perform my job effectively”, indicated that these factors were significantly related to both occupational and general stress among COs, with weaker administrations leading to higher stress [42]. In contrast, one study examining administrative strengths, using an unreported measure, was seen to be not significantly related to job stress, however the relationship was in the expected direction of weaker administrations related to higher stress. These results may have been due to their small sample size [41].

Organizational support for the employee is mainly perceived by the amount of pride an organization shows for the work accomplishments of COs. In addition, organizational support can be seen in policies for equal treatments that target inequalities based on culture, gender, race, and so forth. Two of the included studies reported that organizational support was significantly negatively associated with CO job stress [49,51]. Both studies used the same validated measure and included statements such as “The department takes pride in my accomplishments at work”. Griffin (2006), also using the same scale, examined gender differences in the stress stemming from organizational support and determined that male COs in particular experienced high levels of stress when the organization supported equal treatment policies. This factor was not significantly associated with female COs’ job stress [51].

Perceived organizational justice was also examined using a validated measure [15]. According to Taxman and Gordon (2009), the two aspects of organizational justice are distributive justice (“the outcome of a decision or event”) and procedural justice (“the decision making process that leads to the outcome”) [15]. Their study indicated that both aspects of organizational justice were significantly related to job stress as well as organizational commitment among COs [15].

## Discussion

Workplace stress and burnout among COs can lead to unsafe correctional facilities, high turnover rates, high absenteeism, lower productivity and decreased effectiveness in the workplace as well as negative personal and social outcomes like decreased life satisfaction and work-family conflict. Thus, it is important to acknowledge the organizational stressors that are associated with CO stress in order to establish organizational interventions aimed at preventing and decreasing stress and burnout.

The results from this review indicate that the organizational structure and climate had the most consistent relationship with CO job stress and burnout, a factor not previously identified in the review by Schaufeli and Peeters (2000) [18]. These stressors include: unclear goals and policies, lack of decision making ability, lack of support from the organization and lack of organizational justice. Cooper and Marshall’s (1976) remaining four categories: stressors intrinsic to the job, role in organization, rewards at work and supervisory relationships showed inconsistent findings, which is a different result from Schaufeli and Peeters (2000) previous literature review [18]. It has been suggested elsewhere in the literature on organizational stressors among COs that two of these categories of organizational stressors: rewards at work and relationships with supervisors, are significantly associated with job strain and psychological distress [2,16,52]. Despite the fact that these studies did not measure job stress and burnout directly, job strain and psychological distress have been shown to significantly increase the risk for experiences of job stress and burnout among employees [5,53]. Future research could aim to examine the relationship between these stressors, their impact on job strain and psychological distress and the relationship with job stress and burnout. In addition, future research should also re-examine the organizational stress categories of rewards at work, relationship with supervisors, stressors intrinsic to the job and role within the organization and their relationship, in order to establish more conclusively their relationship to job stress and burnout.

In the broader literature on law enforcement personnel job stress and burnout, it has been indicated that organizational stressors experienced by COs also affect police officers, probation officers and parole officers in a similar way. One review, examining organizational stressors in police officers demonstrated that, similar to COs, supervisory relationships, such as inadequate supervision, and organizational structure and climate, such as organizational justice, significantly impacted police officer job stress [54]. In addition, police officer stress was consistently impacted by stressors intrinsic to the job, role in organization and rewards at work [54]. Another study also demonstrated that lack of organizational support, lack of opportunity for promotion and role conflict also significantly impacted job stress in both COs and police officers [55]. In parole and probation officers, similar organizational stressors and outcomes have been described. Probation officer job stress is demonstrated to be significantly impacted by supervisory support [56]. Both probation and parole officer job stress and burnout is impacted by work overload and inadequate resources [57]. Given the similarities between the various employees within the law enforcement industry, the results presented in this paper may be generalizable to other law enforcement personnel. Future research should continue to examine similarities and differences between law enforcement personnel in order to establish applicability of interventions across this industry.

### Current state of the literature

The studies included in this review had two main strengths. First, they all used validated instruments to measure job stress and burnout as well as the organizational stressors. The use of validated measures for stress and burnout increases the degree to which they measure these constructs. Validated measures, where used, increase the reliability of the results obtained from the eight included studies. In addition, all of the included studies used large samples of mainly male Caucasians which is characteristic of correctional facilities [41,50]. This increases the generalizability of the results obtained to the larger population of COs.

There are several limitations of the included studies. First, all of the studies were cross-sectional in design and were therefore unable to establish causal relationships between the variables. Second, in addition to not using the same measure for job stress and burnout, the studies did not use the same measure or same response for organizational stressors. In addition, the subjective nature of the measures allows for self-reporting and recall biases in the data obtained. Lastly, the studies used samples that were mainly male. Therefore, to the extent that increasing numbers of women serve as COs, special consideration may be needed for women (Table 2).

It will be useful if longitudinal studies are conducted and standard objective measures be used in future research examining organizational stressors in COs. In addition, future research should examine the differences, if any, between the various types of correctional facilities in order to determine whether the sources of stress impact employees from different facilities in the same way.

### Limitations of the review

Although there are a number of strengths outlined above, the current review also has limitations. First, six of the eight included studies in this literature review originate from the United States of America. As such, the results outlined in this paper may not be generalizable to COs in other countries.

Second, because this review only examined organizational stressors for COs, the results presented may not be generalizable to other employees within the correctional organization. Several studies not included in this particular review illustrated that regardless of position, there were certain commonalities between correctional employees such as exposure to the same environment [58]. On the other hand, as previously mentioned, several studies also argue for the fact that there are differences between COs and other employees within the correctional facility. For example, COs have: less input into administrative decisions than supervisors [59], increased risk of inmate violence [60], the central task of inmate supervision [58], routine responsibilities and the least autonomy [61].

Finally, all of the studies included in this review used samples of COs who were employed in public rather than private correctional institutions. It has been noted that the organizational environment in private facilities is different from public facilities [61]. As such, the results obtained may not be generalizable to correctional officers in private correctional facilities. Similarly, this review did not examine COs by facility type. Although the literature suggests that this may not be an important distinction [19], there may be differences that have not yet been identified.

It is recommended that future studies conduct comparative assessments of: organizational stressors in COs internationally, across types of facilities as well as differences in the impact of these stressors across the range of correctional positions.

### Implications for practice

There are at least five groups that are able to benefit from a mentally healthy workforce: the public, the employers, the workers and their families and insurance companies [3]. Within correctional facilities, the inmates also benefit from a healthy workforce. Reducing worker stress and burnout has the ability to increase morale, productivity, efficiency, effectiveness and general well-being. It also has the potential to reduce early retirement, worker’s compensation claims, on-the-job accidents, civil liabilities for counter-productive behavior and negative attention from the media [62]. In addition, as mentioned by the World Health Organization (1998), benefits of reduced employee stress for the correctional organization, specifically, include improved security and safety, positive staff-inmate relationships, retention of COs, less absence due to illness and greater cost effectiveness [63].

Based on the results of the current literature review, interventions should aim to increase and improve communication between management and staff thereby improving the organizational structure and climate of the correctional facility and reducing the risk of job stress and burnout [1,15,64]. Specifically, COs would benefit from clearly defined goals and guidelines, increased participation in decision making, increased sense of support from the organization and increased organizational justice. Increasing the communication between management and COs can be accomplished in several ways. First, management can provide COs with a clearer written description of the goals and policies of the correctional facilities [65,66]. Second, organizations can increase the number of collaborative meetings between management and COs, thereby facilitating the ability of COs to participate in decision making within the organization [65,66]. Organizations can increase the transparency of the processes and factors involved in the decisions that they make thereby increasing the COs' perception of organizational justice and that all decisions that are made by management are fair [65,66]. Finally, organizations can increase the support of the COs by formally recognizing COs' contributions to the workplace [66]. Future research should examine the effectiveness of these interventions for reducing job stress and burnout among COs.

## Conclusion

The results of this review indicate that organizational stressors are associated with job stress and burnout in COs within adult correctional facilities. Specifically, the organizational structure and climate was significantly associated with CO job stress and burnout. The other categories of organizational stressors including: stressors intrinsic to the job, role within the organization, rewards at work and supervisory support showed inconsistent findings. Future research should continue to examine these stressors among COs. Given that the organizational structure and climate was significantly associated with CO job stress and burnout, organizational interventions should aim to improve the communication between management and COs. For example, organizations can increase collaborative meetings between management and COs. Reducing CO job stress and burnout can lead to positive outcomes not only for the individual, but the organization as well (e.g. decreased absenteeism). Future research should continue to examine the effectiveness of increasing the communication between management and COs in reducing job stress and burnout.

## Appendix

Search TermsDatabase: PsycInfoSearch Terms:(correction* officer*.mp. or prison* guard*.mp. or exp corrections officers or exp prison personnel) and (exp stress or exp chronic stress or exp environmental stress or exp occupational stress or exp psychological stress or exp social stress or exp stress reactions or exp distress or exp organizational crises) limit to “0100 journal” and yr=”1999-Current”Database: MedlineSearch Terms:(correction* officer*.mp. or prison* guard*.mp. or prison* personnel*.mp.) and (exp stress, psychological or stress* reaction*.mp. or distress*.mp. or organiz* crises*.mp. or job* stress*.mp. or job* stress*.mp. or burnout*.mp. or chronic* stress*.mp. or environment* stress*.mp. or occupation* stress*.mp. or social* stress*.mp. or stress*.mp.) limit to yr=”1999-Current”Database: Criminal Justice AbstractsSearch Terms:(exp correctional personnel or correction* officer* or prison* guard*) and (exp stress or exp stress psychology or exp job stress or exp burnout or chronic* stress* or environment* stress* or occupation* stress* or social* stress* or stress* reaction* or distress* or organization* crises*)Database: Sociological Abstracts(su(corrections officers) or all(“prison guard*”) or all(“prison personnel*”) or all(“correction* officer*”)) and (all(stress*) or all(burnout*))Googlecorrectional officer mental health, international correctional officer mental health, international correctional staff mental health, international prison management guidelines, international prison staff stress management programs, international correction officer guidelines, international correction officer, international corrections employment guidelines, international prison employee guidelinesGovernment and International OrganizationsNational Institute of Justice (U.S. Department of Justice)Correctional Services CanadaInternational Corrections and Prisons AssociationWorld Health OrganizationNational Institute of CorrectionsThe Corrections Connection (corrections.com)

## Competing interests

The authors declare that they have no competing interests.

## Authors’ contributions

CF led the conception, design, data acquisition, analysis and interpretation of the data. ES collaborated on the design, data acquisition and analysis. JH collaborated on the analysis and interpretation. SB collaborated on the design and data acquisition. CSD collaborated on the conception, design and acquisition of data, and supervised the data analysis and interpretation. All authors read and approved the final manuscript.

## Pre-publication history

The pre-publication history for this paper can be accessed here:

http://www.biomedcentral.com/1471-2458/13/82/prepub

## Supplementary Material

Additional file 1 **Quality Assessment_Organizational Stressors Associated with Job Stress and Burnout in Correctional Officers doc.** The additional file contains the quality checklist criteria that were used to determine the quality of the papers being analyzed for the systematic review. Scores of each article are displayed as well as the quality checklist items that were adapted from Stergiopoulos et al. (2011). Click here for file

## References

[B1] Cooper CL Marshall J Occupational sources of stress: a review of the literature relating to coronary heart disease and mental ill health J Occup Psychol 1976 49 11 28 10.1111/j.2044-8325.1976.tb00325.x

[B2] Bourbonnais R Malenfant R Vezina M Jauvin N Brisson I Les caracteristiques du travail et la sante des agents en service de detention Revue Epidemiologique Sante Publique 2005 53 127 142 10.1016/S0398-7620(05)84583-3 16012372

[B3] Dewa CS McDaid D Ettner SL An international perspective on worker mental health problems: who bears the burden and how are costs addressed? Can J Psychiatry 2007 52 6 346 356 17696020 10.1177/070674370705200603

[B4] Cullen FT Link BG Wolfe NT Frank J The social dimensions of correctional officer stress Justice Quarterly 1985 2 4 505 533 10.1080/07418828500088711

[B5] Maslach C Schaufeli WB Leiter MP Job burnout Annual Revue of Psychology 2001 52 397 422 10.1146/annurev.psych.52.1.397 11148311

[B6] Parker DF DeCotiis TA Organizational determinants of job stress Organ Behav Hum Perform 1983 32 160 177 10.1016/0030-5073(83)90145-9

[B7] Cooper CL Cartwright S Healthy mind, healthy organization - A proactive approach to occupational stress Human Relations 1994 47 4 455 471 10.1177/001872679404700405

[B8] Brief AP Weiss HM Organizational behavior: affect in the workplace Annu Rev Psychol 2002 53 297 307 10.1146/annurev.psych.53.100901.135156 11752487

[B9] Correctional Service Canada Organizationhttp://www.csc-scc.gc.ca/text/organi-eng.shtml

[B10] Correctional officer - Group & level CX-01 http://www.csc-scc.gc.ca/text/carinf/correctional-eng.shtml

[B11] Correctional officershttp://www.bls.gov/ooh/protective-service/correctional-officers.htm

[B12] Corrections officerhttp://www.corrections.govt.nz/careers/opportunities-at-corrections/prison-services-jobs/corrections-officer.html

[B13] World Health Organization Moller L, Stover H, Jurgens R, Gartherer A, Nikogosian H Health in prisons: A WHO guide to the essentials in prison health 2007 Copenhagen, Denmark: World Health Organization

[B14] Lambert EG Hogan NL Altheimer I An exploratory examination of the consequences of burnout in terms of life satisfaction, turnover intent, and absenteeism among private correctional staff The Prison J 2010 90 1 94 114 10.1177/0032885509357586

[B15] Taxman FS Gordon JA Do fairness and equity matter? An examination of organizational justice among correctional officers in adult prisons Crim Justice Behav 2009 36 7 695 711 10.1177/0093854809335039

[B16] Bourbonnais R Jauvin N Dussault J Vezina M Psychosocial work environment, interpersonal violence at work and mental health among correctional officers Int J Law Psychiatry 2007 30 355 368 10.1016/j.ijlp.2007.06.008 17681602

[B17] Crank J Regoli R Hewitt J Culbertson R Institutional and organizational antecedents of role stress, work alienation and anomie among police executives Crim Justice Behav 1995 22 152 171 10.1177/0093854895022002004

[B18] Schaufeli WB Peeters MCW Job stress and burnout among correctional officers: a literature review Int J Stress Manage 2000 7 1 19 48 10.1023/A:1009514731657

[B19] Dowden C Tellier C Predicting work-related stress in correctional officers: a meta-analysis J Crim Justice 2004 32 31 47 10.1016/j.jcrimjus.2003.10.003

[B20] Gerstein LH Topp CG Correll G The role of the environment and person when predicting burnout among correctional personnel Crim Justice Behav 1987 14 3 352 369 10.1177/0093854887014003006

[B21] Carlson JR Thomas G Burnout among prison case workers and corrections officers Journal of Offender Rehabilitation 2008 43 3 19 34

[B22] Paoline EA Lambert E Hogan NL A calm and happy keeper of the keys: The impact of ACA views, relations with co-workers, and policy views on the job stress and job satisfaction of correctional staff The Prison Journal 2006 86 2 182 205 10.1177/0032885506287819

[B23] Hepburn JR Knepper PE Correctional officers as human service workers: the effect on job satisfaction Justice Quarterly 1993 10 2 315 337 10.1080/07418829300091841

[B24] Robinson D Porporino FJ Simourd L The influence of educational attainment on the attitudes and job performance of correctional officers Crime Delinquen 1997 43 1 60 77 10.1177/0011128797043001004

[B25] Manual do agente penitenciario http://www.depen.pr.gov.br/arquivos/File/manual_agente_pen.pdf

[B26] Becoming a prison officerhttp://www.justice.gov.uk/jobs/prisons/on-offer/prison-officer

[B27] The training institute for prison and probation serviceshttp://www.rskk.fi/inenglish

[B28] Personnels de surveillance: Des carrieres au services de la societehttp://www.metiers.justice.gouv.fr/presentation-des-metiers-10070/les-metiers-de-ladministration-penitentiaire-10072/personnels-de-surveillance-11803.html

[B29] La organizacion de la Secretaria General de Instituciones Penitenciarias http://www.institucionpenitenciaria.es/web/portal/administracionPenitenciaria/organizacion/

[B30] Prison types & general informationhttp://www.bop.gov/locations/institutions/index.jsp

[B31] Terms & definitions: Correctionshttp://bjs.ojp.usdoj.gov/index.cfm?ty=tdtp&tid=1

[B32] Les structures penitentiaireshttp://www.justice.gouv.fr/prison-et-reinsertion-10036/ladministration-penitentiaire-10037/les-structures-penitentiaires-14557.html

[B33] World factbook of criminal justice systemshttp://bjs.ojp.usdoj.gov/index.cfm?ty=pbdetail&iid=1435

[B34] Edwards J An examination of competing versions of the person-environment fit approach to stress Acad Manage J 1996 39 292 339 10.2307/256782

[B35] Karasek RA Job demands, job decision latitude, and mental strain: implications for job redesign Adm Sci Q 1979 24 2 285 308 10.2307/2392498

[B36] Seigrist J Adverse health effects of high-effort/low-reward conditions J Occup Health Psychol 1996 1 1 27 41 9547031 10.1037//1076-8998.1.1.27

[B37] Johnson S Cooper CL The construct validity of the ASSET stress measure Stress and Health 2003 19 99 113

[B38] Johnson S Cooper CL Cartwright S Donald I Taylor P Millet C The experience of work-related stress across occupations J Manag Psychol 2005 20 2 178 187 10.1108/02683940510579803

[B39] French JRP Caplan RD Marrow AJ Organizational stress and individual strain The Failure of Success 1973 AMACOM, New York 30 66

[B40] Moon B Maxwell SR The sources and consequences of corrections officers' stress: a South Korean example J Crim Justice 2004 32 359 370 10.1016/j.jcrimjus.2004.04.006

[B41] Castle TL Satisfied in jail? Exploring the predictors of job satisfaction among jail officers Crim Justice Rev 2008 33 1 48 63 10.1177/0734016808315586

[B42] Castle TL Martin JS Occupational hazard: predictors of stress among jail correctional officers Am J Crim Justice 2006 31 1 65 80 10.1007/BF02885685

[B43] Finn P Correctional officer stress: a cause for concern and additional help Federal Probation 1998 62 2 65 74

[B44] Lambert E Paoline EA Take this job and shove it: an exploratory study of turnover intent among jail staff J Crim Justice 2010 38 139 148 10.1016/j.jcrimjus.2010.01.002

[B45] Stergiopoulos E Cimo A Cheng C Bonato S Dewa CS Interventions to improve work outcomes in work-related PTSD: a systematic review BMC Public Health 2011 11 838 10.1186/1471-2458-11-838 22040066 PMC3219578

[B46] Schuler RS Definition and conceptualization of stress in organizations Organ Behav Hum Perform 1980 25 184 215 10.1016/0030-5073(80)90063-X

[B47] Motowidlo SJ Packard JS Manning MR Occupational stress: its causes and consequences for job performance J Appl Psychol 1986 71 4 618 629 3804934

[B48] Summerlin Z Oehme K Stern N Valentine C Disparate levels of stress in police and correctional officers: preliminary evidence from a pilot study on domestic violence J Hum Behav Soc Environ 2010 20 762 777 10.1080/10911351003749169

[B49] Armstrong GS Griffin ML Does the job matter? Comparing correlates of stress among treatment and correctional staff in prisons J Crim Justice 2004 32 577 592 10.1016/j.jcrimjus.2004.08.007

[B50] Neveu JP Jailed resources: conservation of resources theory as applied to burnout among prison guards J Organ Behav 2007 28 21 42 10.1002/job.393

[B51] Griffin ML Gender and stress: a comparative assessment of sources of stress among correctional officers J Contemporary Crim Justice 2006 22 1 4 25

[B52] Lavigne E Bourbonnais R Psychosocial work environment, interpersonal violence at work and psychotropic drug use among correctional officers Int J Law Psychiatry 2010 33 122 129 10.1016/j.ijlp.2009.12.005 20042239

[B53] Felton JS Burnout as a clinical entity: its importance in health care workers Occup Med 1998 48 4 237 250 10.1093/occmed/48.4.237 9800422

[B54] Abdollahi MK Understanding police stress research J Forensic Psychol P 2002 2 2 1 24 10.1300/J158v02n02_01

[B55] Anson RH Johnson B Anson NW Magnitude and source of general and occupational-specific stress among police and correctional officers Journal of Offender Rehabilitation 1997 25 1/2 103 113

[B56] Brown PW Probation officer burnout Federal Probation 1987 51 17

[B57] Whitehead J Lindquist C Job stress and burnout among probation/parole officers: perceptions and causal factors Int J Offender Ther Comp Criminol 1985 29 2 109 119 10.1177/0306624X8502900204

[B58] Lambert EG Hogan NL Griffin ML The impact of distributive and procedural justice on correctional staff job stress, job satisfaction and organizational commitment Journal of Criminal Justice 2007 35 6 644 656 10.1016/j.jcrimjus.2007.09.001

[B59] Hogan NL Lambert E Jenkins M Wambold S The impact of occupational stressors on correctional staff organizational commitment: a preliminary study J Contemporary Crim Justice 2006 22 1 44 62 10.1177/1043986205285084

[B60] Kienan G Malach-Pines A Stress and burnout among prison personnel Crim Justice Behav 2007 34 3 380 398 10.1177/0093854806290007

[B61] Lambert EG Hogan NL Paoline EA Stevenson MT I want to know and I want to be a part of it: the impact of instrumental communication and integration on private prison staff Journal of Applied Security Research 2008 3 2 205 229 10.1080/19361610802135938

[B62] Finn P Tomz JE Developing a law enforcement stress program for officers and their families Issues and Practices in Criminal Justice 1996 Washington, DC: National Institute of Justice

[B63] World Health Organization Mental health promotion in prisons: Report on a WHO meeting 1998 The Hague, Netherlands: World Health Organization

[B64] Dollard MF Winefield AH Organizational response to recommendations based on a study of stress among correctional officers Int J Stress Manage 1994 1 1 81 101 10.1007/BF01857284

[B65] Dussault J Jauvin N Vezina M Bourbonnais R Preventing violence among employees of the same work organization: Evaluation of a participatory intervention Collection Etudes et Recherches 2012 Montreal: IRSST Publications

[B66] Bourbonnais R Jauvin N Dussault J Vezina M Biron C, Karanika-Murray M, Cooper CL Evaluation of an intervention to prevent mental health problems among correctional officers Improving Organizational Interventions for Stress and Well-being: Addressing Process and Context 2012 New York: Routledge

