# Supplementary material for: Organizational stressors associated with job stress and burnout in correctional officers: a systematic review
Source: BMC Public Health. 2013 Jan 29;13:82. doi: 10.1186/1471-2458-13-82 (PMC3564928; doi:10.1186/1471-2458-13-82)
Supplement: Additional file 1 — Quality Assessment_Organizational Stressors Associated with Job Stress and Burnout in Correctional Officers doc. The additional file contains the quality checklist criteria that were used to determine the quality of the papers being analyzed for the systematic review. Scores of each article are displayed as well as the quality checklist items that were adapted from Stergiopoulos et al. (2011). [file 1471-2458-13-82-S1.pdf]

Quality Assessment Checklist

Reference	1	2	3	4	5	6	7	8	TOTAL
Bourbonnais et al., 2005	1	1	1	1	1	1	1	1	8
Taxman & Gordon, 2009	0	1	1	1	1	1	1	1	7
Summerlin et al., 2010	0	1	1	0	0	0	1	1	4
Neveu, 2007	1	1	1	1	1	1	1	1	8
Moon & Maxwell, 2004	1	1	1	0	1	1	1	0	6
Lavigne & Bourbonnais, 2010	1	1	1	1	1	1	1	1	8
Griffin, 2006	0	1	1	0	1	1	1	1	6
Castle & Martin, 2006	0	1	1	0	1	1	1	1	6
Bourbonnais et al., 2007	1	1	1	1	1	1	1	1	8
Armstrong & Griffin, 2004	0	1	1	1	1	1	1	1	7
Castle, 2008	0	1	1	1	1	1	1	1	7

Quality Assessment Criterion:
1 = Study's sample is representative of the population of corrections officers
2 = Methods of data collection are described
3 = Validated measures are used
4 = Confounders are mentioned and accounted for in the analysis
5 = The statistical method used is appropriate for the outcome studied
6 = Statistical significance of the association(s) are tested and relevant parameters are presented
7 = The author(s) answered their research question
8 = There is a discussion of study limitations, including biases, and the way in which the study may have been affected as a result
